# *In vitro* activity of cefepime/zidebactam against *Klebsiella pneumoniae* carrying *bla*_KPC_ variants conferring resistance to ceftazidime/avibactam

**DOI:** 10.1093/jacamr/dlag026

**Published:** 2026-03-02

**Authors:** Carlo Tascini, Steven H Marshall, Robert A Bonomo, Paolo Gaibani

**Affiliations:** Department of Medicine, Università Degli Studi di Udine, Udine, Italy; Infectious Diseases Unit, Azienda Sanitaria Universitaria Friuli Centrale, Udine, Italy; Louis Stokes Cleveland Department of Veterans Affairs Medical Center, Cleveland, OH, USA; Department of Molecular Biology and Microbiology, Case Western Reserve University School of Medicine, Cleveland, OH, USA; CWRU-Cleveland VAMC Center for Antimicrobial Resistance and Epidemiology (Case VA CARES), Cleveland, OH, USA; Research Service and GRECC, Louis Stokes Cleveland Department of Veterans Affairs Medical Center, Cleveland, OH, USA; Departments of Medicine, Pharmacology, Molecular Biology and Microbiology, Biochemistry, Proteomics and Bioinformatics, Case Western Reserve University School of Medicine, Cleveland, OH, USA; Department of Diagnostic and Public Health, Microbiology Section, Verona University, Verona, Italy; Microbiology and Virology Unit, Azienda Ospedaliera Universitaria Integrata Di Verona, Verona, Italy

## Abstract

**Objectives:**

Resistance to ceftazidime/avibactam among KPC-producing *Klebsiella pneumoniae* (KPC-*Kp*) is often due to mutations within the *bla*_KPC_ gene, determining a widespread occurrence of novel variants. Against KPC-*Kp* carrying novel *bla*_KPC_ variants, further therapeutic agents are needed.

**Methods:**

We evaluated the *in vitro* activity of cefepime/zidebactam against 21 KPC-*Kp* clinical isolates carrying different *bla*_KPC_ variants showing different antimicrobial susceptibility patterns to ceftazidime/avibactam, and compared it with the *in vitro* activity of cefepime/enmetazobactam. WGS was performed to identify antimicrobial resistance genes associated with ceftazidime/avibactam resistance. Analysis of porins and PBP-2 sequences was performed by manual alignment. Antimicrobial susceptibility testing to cefepime, cefepime/enmetazobactam and cefepime/zidebactam was performed by MIC test strips.

**Results:**

We selected a total of 21 ceftazidime/avibactam susceptible (*n* = 9) or resistant (*n* = 12) strains. Genomic analysis revealed that all ceftazidime/avibactam-resistant KPC-*Kp* carried mutations within *bla*_KPC_ variants (*bla*_KPC-31_, *bla*_KPC-14_, *bla*_KPC-33_, *bla*_KPC-93,_  *bla*_KPC-203_, *bla*_KPC-205_, *bla*_KPC-49_ and *bla*_KPC-167_), whereas susceptible strains carried *bla*_KPC-3_ and *bla*_KPC-2_ alleles. Overall, 42.85% (9/21) and 4.76% (1/21) of KPC-*Kp* harboured, respectively, a truncated OmpK35 or OmpK36 porin. PBP-2 analysis showed that all KPC-*Kp* carried WT enzymes, whereas one isolate carried a V521M substitution (valine→methionine). Cefepime/zidebactam (median 0.38 mg/L, IQR 0.222–0.5 mg/L) exhibited greater antibacterial activity (*P* < 0.0001) than cefepime alone and cefepime/enmetazobactam against ceftazidime/avibactam-susceptible KPC-*Kp*, whereas it exhibited no statistically significant difference (*P* = 0.4621) in antibacterial activity compared with cefepime/enmetazobactam against ceftazidime/avibactam-resistant strains carrying *bla*_KPC_ variants. Also, we observed that cefepime/zidebactam exhibited greater antibacterial activity (*P* < 0.001) against KPC-*Kp* strains carrying the mutated *bla*_KPC_ gene than against isolates harbouring the WT *bla*_KPC_ gene.

**Conclusions:**

Cefepime/zidebactam provided potent *in vitro* results against KPC-*Kp* due to *bla*_KPC_ variants, supporting its clinical utility for the treatment of infections due to ceftazidime/avibactam-resistant strains. Also, we demonstrated that zidebactam was not influenced by different *bla*_KPC_ variants.

## Introduction

Carbapenemase-producing Enterobacterales (CPE) are a major clinical concern due to the limited therapeutic options and to the difficult-to-treat infections caused by these microorganisms.^1^ The development of new β-lactam/β-lactamase inhibitor combinations (βL-βLICs) initially overcame the limited antimicrobial options available against KPC-bearing strains and enabled novel strategies to limit the devastating impact of CPE worldwide.^2^ However, the recent emergence of strains resistant to novel βL-βLICs has been observed in different countries, thus mitigating clinical usage of these novel molecules.^3,4^ In this context, an urgent need exists for novel molecules or innovative therapeutic approaches to counteract novel resistance among CPE.^2^

Resistance to novel βL-βLICs is due to a variety of different mechanisms of action.^3,5^ Resistance to ceftazidime/avibactam is often linked to mutations in the *bla*_KPC_ gene, which may lead to a ‘reversion’ phenotype characterized by restored susceptibility to carbapenems and novel agents active against ESBL-producers, such as cefepime/enmetazobactam.^5–9^ Recently, a novel combination, cefepime/zidebactam, has been proposed for the treatment of CPE.^8^ Cefepime is an advanced fourth-generation cephalosporin that exerts its bactericidal action by inhibiting PBP-3. Zidebactam is a bicyclo-acyl hydrazide β-lactam enhancer with a dual action: inhibition of class A and C β-lactamases and enhancement of the antibacterial activity of cefepime through specific binding to PBP-2.^8^ The aim of this study was to evaluate the *in vitro* activity of cefepime/zidebactam against ceftazidime/avibactam-resistant *Klebsiella pneumoniae* (KPC-*Kp*) isolates carrying *bla*_KPC_ variants with or without PBP-2 mutation, and compare it with the *in vitro* activity of cefepime/enmetazobactam.

## Materials and methods

In this study, a total of 21 KPC-producing *K. pneumoniae* strains were selected on the basis of antimicrobial susceptibility profiles to ceftazidime/avibactam. The KPC-producing *K. pneumoniae* isolates were selected from strain collections isolated from patients recovered in different hospitals in Italy. The selection comprised all consecutive blood culture isolates collected in 2022/23 that were KPC producers. Duplicate KPC-producing *K. pneumoniae* isolates collected from the same patient were excluded.

Antimicrobial susceptibility testing was performed using Sensititre Gram-negative MDRGN1F (Thermofisher, MS, USA); MICs of cefepime, cefepime/enmetazobactam, and cefepime/zidebactam were determined by MIC TestStrip (Liofilchem, Italy), and results were interpreted following the EUCAST (cefepime and cefepime/enmetazobactam; https://www.eucast.org/clinical_breakpoints) and CLSI (cefepime/zidebactam; https://www.nih.org.pk/wp-content/uploads/2021/02/CLSI-2020.pdf) clinical breakpoints. MICs determined as equal or superior to the limit of the Strips were considered at the high value. Comparison between MICs of different antibiotics was performed using Student’s *t*-test analysis implemented in GraphPad Prism v.10.1.11 (San Diego, CA, USA).

WGS was performed as previously described [6]. Briefly, paired-end libraries were prepared using the Illumina DNA Prep Kit and sequenced on the Illumina MiSeq system (Illumina, CA, USA). Read quality was evaluated with FastQC v0.12.1, and genome assembly was carried out using SPAdes v3.15.2 (https://github.com/ablab/spades). MLST and resistome analysis were performed using AMRFinderPlus (https://github.com/ncbi/amr). Analysis of PBP-2 was performed by aligning the sequence obtained from each genome of KPC-*Kp* included in this study against the genome of *K. pneumoniae* ssp. *pneumoniae* HS11286 used as reference.^10^

The draft genome assemblies of the KPC-*Kp* strains included in this study have been deposited in the NCBI BioSample database under SubmissionID SAMN55289090-SAMN55289110 and BioProject ID PRJNA1421143

## Results and discussion

Overall, we selected a total of 21 KPC-*Kp* grouped as susceptible (*n* = 9) or resistant (*n* = 12) to ceftazidime/avibactam. Genotypic characteristics of the KPC-*Kp* strains included in this study are shown in Table 1. Complete genomic features of KPC-*Kp* strains included in this study are shown in Table S1 (available as Supplementary data at *JAC-AMR* Online). Genomic analysis showed that 11 out of 21 (52%) isolates belonged to the clonal complex (CC) 101 (including ST101 and ST1685), 9 (43%) to CC512 (including ST512 and ST1519), and 1 (5%) to ST307. Analysis of serotype based on O antigen sequences showed that O1/O2v2 and O1/O2v1 alleles were present, respectively, in 9 (43%) and 11 (52%) out of 21 KPC-*Kp* strains, whereas 1 isolate harboured the OL102 serotype. Analysis of the K antigen demonstrated that allele K17 was observed in 11 (52%) KPC-*Kp*, allele K107 was carried in 9 (43%), and K102 was present in 1 of the KPC-*Kp* strains [6].

**Table 1.** Genotypic characteristics of KPC-producing *Klebsiella pneumoniae* included in this study

Isolate	MLST	Serotype		PBP-2	Porins		Antimicrobial resistance genes to β-lactams	
	ST	O	K		OmpK35 (359 aa)	OmpK36 (367 aa)	Carbapenemase	ESBL
1	512	O1/O2v2	K107	WT	Truncated	135GD_Ins	*bla*_KPC-31_	*bla*_TEM-1_, *bla*_SHV-11_
2	512	O1/O2v2	K107	WT	Truncated	135GD_Ins	*bla*_KPC-31_	*bla*_TEM-1_, *bla*_SHV-11_
3	512	O1/O2v2	K107	WT	Truncated	135GD_Ins	*bla*_KPC-31_	*bla*_TEM-1_, *bla*_SHV-11_
4	512	OL102	K107	WT	Truncated	135GD_Ins	*bla*_KPC-3_	*bla*_TEM-1_, *bla*_SHV-11_, *bla*_OXA-10_
5	101	O1/O2v1	KL17	WT	WT	135GD_Ins	*bla*_KPC-3_	*bla*_TEM-1_, *bla*_SHV-1_, *bla*_OXA-1_, *bla*_CTX-M-15_
6	1519	O1/O2v2	K107	WT	Truncated	135GD_Ins	*bla*_KPC-3_	*bla*_TEM-1_, *bla*_SHV-11_
7	512	O1/O2v2	K107	WT	Truncated	135GD_Ins	*bla*_KPC-205_	*bla*_TEM-1_, *bla*_SHV-11_
8	101	O1/O2v1	K107	WT	WT	135GD_Ins	*bla*_KPC-203_	*bla*_SHV-1_
9	101	O1/O2v1	K107	WT	WT	135GD_Ins	*bla*_KPC-3_	*bla*_TEM-1_, *bla*_SHV-1_
10	101	O1/O2v1	K107	WT	WT	135GD_Ins	*bla*_KPC-3_	*bla*_TEM-1_, *bla*_SHV-1_
11	307	O1/O2v2	K102	WT	WT	truncated	*bla*_KPC-2_	*bla*_TEM-1_, *bla*_SHV-28_, *bla*_OXA-1_, *bla*_CTX-M-15_
12	512	O1/O2v2	K107	V521M	Truncated	135GD_Ins	*bla*_KPC-3_	*bla*_TEM-1_, *bla*_SHV-11_, *bla*_OXA-10_, *bla*_CMY-16_
13	101	O1/O2v1	K17	WT	WT	135GD_Ins	*bla*_KPC-93_	*bla*_TEM-1_, *bla*_SHV-1_
14	101	O1/O2v1	K17		WT	135GD_Ins	*bla*_KPC-2_	*bla*_TEM-1_, *bla*_SHV-1_
15	512	O1/O2v2	K107	WT	Truncated	135GD_Ins	*bla*_KPC-49_	*bla*_TEM-1_, *bla*_SHV-11_
16	101	O1/O2v1	K17	WT	WT	136TDT_Ins	*bla*_KPC-2_	*bla*_SHV-1_
17	101	O1/O2v1	K17	WT	WT	136TDT_Ins	*bla*_KPC-33_	*bla*_SHV-1_
18	101	O1/O2v1	K17	WT	WT	136TDT_Ins	*bla*_KPC-14_	*bla*_SHV-1_
19	1685	O1/O2v1	K17	WT	WT	136TDT_Ins	*bla*_KPC-14_	*bla*_TEM-1_, *bla*_SHV-1_, *bla*_OXA-1_, *bla*_CTX-M-15_
20	101	O1/O2v1	K17	WT	WT	135GD_Ins	*bla*_KPC-3_	*bla*_TEM-1_, *bla*_SHV-1_
21	512	O1/O2v2	K107	WT	Truncated	135GD_Ins	*bla*_KPC-167_	*bla*_TEM-1_, *bla*_SHV-1_

aa, amino acids; Ins, insertion.

Resistome analysis of the genomes of KPC-*Kp* included in this study was performed in order to identify the molecular mechanisms related to resistance to ceftazidime/avibactam and, in particular, substitution within the *bla*_KPC_ gene. Resistome analysis results showed that 12 of the 21 (57%) KPC-*Kp* harboured *bla*_KPC_ variants associated with ceftazidime/avibactam resistance, whereas 9 (43%) KPC-*Kp* carried WT *bla*_KPC_ (i.e. *bla*_KPC-2_ and *bla*_KPC-3_) alleles. In particular, *bla*_KPC_ variants included *bla*_KPC-31_ (*n* = 3), *bla*_KPC-14_ (*n* = 2), *bla*_KPC-33_ (*n* = 1), *bla*_KPC-93_ (*n* = 1), *bla*_KPC-203_ (*n* = 1), *bla*_KPC-205_ (*n* = 1), *bla*_KPC-49_ (*n* = 1) and *bla*_KPC-167_ (*n* = 1), whereas *Kp* strains carrying WT alleles included *bla*_KPC-3_ (*n* = 7) and *bla*_KPC-2_ (*n* = 3). Structural analysis of KPC variants associated with resistance to ceftazidime/avibactam among *Kp* strains included in this study showed that mutations occurred in the three hotspot regions (Table S2). In detail, five variants (KPC-49, KPC-31, KPC-31, KPC-167 and KPC-203) harboured mutations (i.e. S163R, D179Y, EL deletion at position 165–166) within the omega loop (residues 163 to 179); a single variant (KPC-14) harboured a mutation (i.e. GT deletion at position 242–243) within the 237–243-loop; and four variants (KPC-93, KPC-167, KPC-203 and KPC-205) harboured insertions (i.e. NRAPN at position 269, iDDKYSE at position 269 and VYTRAPMLA at position 261) within the loop 266–275.

Analysis of porin genes showed that 42.85% (9/21) of KPC-*Kp* included in this study harboured a truncated OmpK35, whereas only one isolate (4.76%) carried a truncated OmpK36 porin (Table 1). At the same time, 76.19% (16/21) of KPC-*Kp* carried a glycine-aspartic acid insertion at position 135, and 19.04% (4/21) harboured a threonine-aspartic acid-threonine insertion at position 136 within OmpK36. Complete genetic characteristics of antimicrobial genes found within genomes included in this study are shown in Table 1 and Table S3. In order to evaluate the presence of mutations within the target of zidebactam (i.e. PBP-2), sequence genome alignment was performed against the genome of *Klebsiella pneumoniae* ssp*. pneumoniae* HS11286, used as reference. PBP-2 analysis showed that all KPC-*Kp* genomes included in this study carried a WT enzyme, although one isolate exhibited a substitution at position 521 (valine→methionine) associated with a higher MIC of cefepime/zidebactam (i.e. MIC = 1 mg/L) relative to the remaining isolates and compared with other KPC-*Kp* strains.

Phenotypic results demonstrated that all KPC-*Kp* strains included in this study were resistant to cefepime, whereas resistance to ceftazidime/avibactam was associated with mutations within the *bla*_KPC_ gene (Table S3). Ceftazidime/avibactam-resistant strains due to mutations within the *bla*_KPC_ gene exhibited restored susceptibility to carbapenems and cefepime/enmetazobactam, as previously reported.

**Figure 1.**
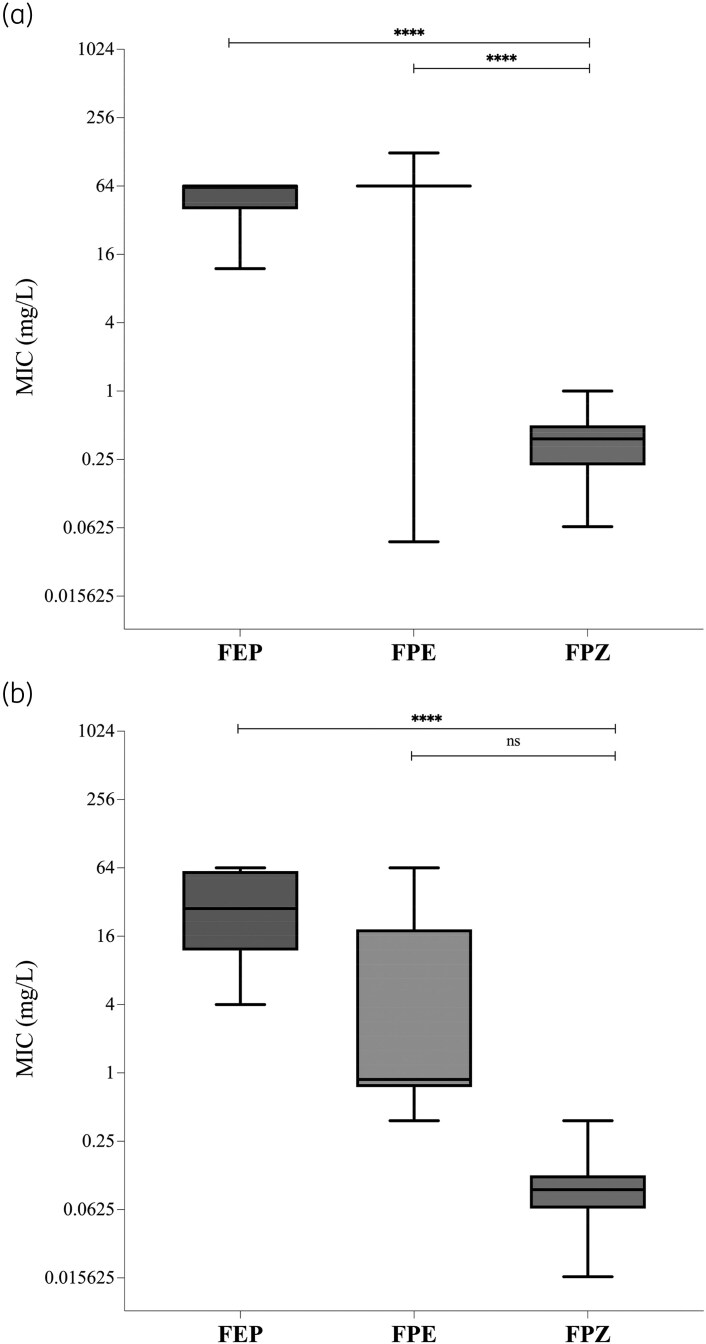
MICs of cefepime (FEP), cefepime/enmetazobactam (FPE) and cefepime/zidebactam (FPZ) against ceftazidime/avibactam-susceptible (a) and -resistant (b) *Klebsiella pneumoniae* clinical strains included in this study.

Our results demonstrated that cefepime/zidebactam exhibited greater antibacterial activity (*P* < 0.0001) than cefepime alone and cefepime/enmetazobactam against all KPC-*Kp* strains included in this study (Figure S1). In particular, we observed that cefepime/zidebactam (median 0.38 mg/L, IQR 0.222–0.5 mg/L) exhibited greater antibacterial activity (*P* < 0.0001) than cefepime (median 64 mg/L, IQR 12–64 mg/L) and cefepime/enmetazobactam (median 64 mg/L, IQR 64 mg/L) against ceftazidime/avibactam-susceptible KPC-*Kp* strains carrying a WT *bla*_KPC_ gene (Figure 1a). Of note, cefepime/zidebactam showed greater antibacterial activity (*P* < 0.001) against strains carrying the mutated *bla*_KPC_ gene (median 0.094 mg/L, IQR 0.064–0.125 mg/L) than against isolates harbouring the WT *bla*_KPC_ gene (median 0.38 mg/L, IQR 0.222–0.5 mg/L). However, the antibacterial activity of cefepime/zidebactam (median 0.094 mg/L, IQR 0.064–0.125 mg/L) was not statistically different (*P* = 0.4621) than that of cefepime/enmetazobactam (median 0.875 mg/L, IQR 0.75–18.38 mg/L) against strains carrying the mutated *bla*_KPC_ gene (Figure 1b).

In this study, we demonstrated that cefepime/zidebactam displayed *in vitro* activity against both ceftazidime/avibactam-susceptible and -resistant KPC-*Kp* strains, and was comparable to the *in vitro* activity of cefepime/enmetazobactam. In detail, we observed that zidebactam enhanced the bactericidal activity of cefepime against *K. pneumoniae* carrying different KPC variants associated with different mutations within the three hotspot regions associated with resistance to ceftazidime/avibactam.^11^ Moreover, we observed that cefepime/zidebactam exhibited similar antibacterial activity to cefepime/enmetazobactam against *K. pneumoniae* carrying the mutated *bla*_KPC_ gene.

Our results agree with previous studies that showed cefepime/zidebactam exhibited excellent bactericidal activity against KPC-producing Enterobacterales.^12^ However, data presented in the literature showed discordant results regarding the *in vitro* activity of cefepime/zidebactam against both ESBL (i.e. CTX) and/or class A (i.e. VEB, SME, KPC), class D (i.e. OXA-48-like) and class B (i.e. VIM, IMP, NDM) carbapenemase-producing Enterobacterales.^12–15^ Previous studies demonstrated that resistance to cefepime/zidebactam is observed among 41.3% (38/92) of *bla*_NDM_-carrying *Klebsiella* spp. and 9.09% (2/22) of *bla*_KPC_-carrying *K. pneumoniae* strains.^14,15^ In particular, Hamza and co-workers^15^ reported that resistance to cefepime/zidebactam is associated with KPC variants carrying mutations in the third hotspot region of the *bla*_KPC_ gene, showing that two out of seven KPC-*Kp* strains carrying mutations within the 270-loop were resistant to cefepime/zidebactam. Both studies reported that alterations of OmpK porin composition (i.e. deleterious alterations within OmpK35 or amino acid modifications/insertions within OmpK36) contributed to the reduced susceptibility to cefepime/zidebactam, thus suggesting a key role for the porins in the resistance to this novel combination.^14,15^

Although KPC-*Kp* strains included in this study carried several modifications among OmpK35 and OmpK36 porins, our results demonstrated that no specific modifications were associated with the reduced susceptibility to cefepime/zidebactam. Analysis of PBP-2 sequences obtained from all isolates showed that all strains carried an L insertion at position 276 in comparison with a reference strain, whereas only one KPC-3-producing *K. pneumoniae* carried the substitution of valine for methionine at position 521 associated with a reduced susceptibility to cefepime/zidebactam in comparison with other KPC-*Kp* strains.

In this context, *bla*_KPC_ variants associated with the resistance to ceftazidime/avibactam are often linked to a restored susceptibility to carbapenems (i.e. meropenem), which does not affect the activity of cefepime/zidebactam due to different target attainment. This suggests the clinical utility of cefepime/zidebactam against variants maintaining resistance to both ceftazidime/avibactam and carbapenems.

Nevertheless, we acknowledge that this study has the major limitation of a relatively small number of strains analysed and the limited number of variants analysed. Consequently, further investigation using a large number of clinical strains is fundamental to evaluate the activity of cefepime/zidebactam against all possible circulating variants.

In conclusion, we demonstrate that cefepime/zidebactam exhibits excellent *in vitro* activity against ceftazidime/avibactam-resistant *K. pneumoniae* carrying *bla*_KPC_ variants, and have expanded knowledge regarding the activity of this novel antimicrobial combination against strains carrying WT and mutated *bla*_KPC_ genes. Further *in vivo* studies will be necessary to define the clinical utility of cefepime/zidebactam and for the treatment of ceftazidime/avibactam-resistant KPC-*Kp*.

## Supplementary Material

dlag026_Supplementary_Data

## Data Availability

Data supporting this study will be available upon reasonable request.

## Supplementary data

Figure S1 and Tables S1 to S3 are available as Supplementary data at JAC-AMR Online.
