# Supplementary material for: In vitro activity of cefepime/zidebactam against Klebsiella pneumoniae carrying blaKPC variants conferring resistance to ceftazidime/avibactam
Source: JAC Antimicrob Resist. 2026 Mar 2;8(1):dlag026. doi: 10.1093/jacamr/dlag026 (PMC12950809; doi:10.1093/jacamr/dlag026)
Supplement: dlag026_Supplementary_Data [file dlag026_supplementary_data.docx]

**Table S1.** Genetic characteristics of the KPC-producing *Klebsiella pneumoniae* clinical strains included in this study

| **Strain** | **Macrolides** | **Aminoglycosides** | **Quinolones** | **MULTIDRUG EFFLUX** | **PLASMIDS (IncType)** |
| --- | --- | --- | --- | --- | --- |
|  | | | | |  |
| 1 | mph(A) | aadA2 aac(6')-Ib | gyrA_S83I parC_S80I | emrD oqxA oqxB | ColRNAI, IncFIB(pQil), IncFII(K), IncN, IncX3 |
| 2 |  | aac(6')-Ib | gyrA_S83I parC_S80I | emrD oqxA oqxB | ColRNAI, IncFIB(pQil), IncFII(K), IncN, IncX3 |
| 3 | mph(A) | aph(3')-Ia aac(6')-Ib | gyrA_S83I parC_S80I | emrD oqxA oqxB |  |
| 5 | mph(A) | aph(3')-Ia aph(3'')-Ib aph(6)-Id aadA1,aadA2 | gyrA_S83I parC_S80I | emrD oqxA oqxB | ColRNAI IncA/C2 IncFIB(K) IncFIB(pQil) IncFII(K) IncX3 |
| 6 | mph(E) | armA aph(3'')-Ib aph(6)-Id aadA1 aac(6')-Ib-cr5 | gyrA_D87N,gyrA_S83Y parC_S80I qnrB | emrD oqxA oqxB20 | Col156 Col440II ColRNAI IncFIA(HI1) IncFIB(AP001918) IncFII IncFII(K) IncR IncQ1 |
| 7 |  | aac(6')-Ib | gyrA_S83I parC_S80I | emrD oqxA oqxB | Col(BS512) ColRNAI IncFIB(K) IncFIB(pKPHS1) IncFIB(pQil) IncFII(K) |
| 8 |  | aadA2 | gyrA_S83I parC_S80I | emrD oqxA oqxB | IncFIB(K) IncFIB(pQil) IncFII(K) |
| 9 | msr(E) mph(E) | armA, aadA1, ant(2'')-Ia | gyrA_S83I parC_S80I | emrD oqxA oqxB20 | Col156, Col440II, ColRNAI, IncFIA(HI1), IncFIB(Mar), IncFII(K), IncR |
| 10 | msr(E) mph(E) | armA | gyrA_D87N,gyrA_S83Y parC_S80I qnrB1 | emrD oqxA oqxB20 | Col156 ColRNAI IncFIA(HI1) IncFII(K) IncR |
| 11 | msr(E) mph(E) | armA | gyrA_D87N,gyrA_S83Y parC_S80I qnrB1 | emrD oqxA oqxB20 | Col156 ColRNAI IncFIA(HI1) IncFII(K) IncR |
| 12 |  | aac(6')-Ib-cr5 | gyrA_D87N,gyrA_S83Y parC_S80I qnrB1 | emrD oqxA oqxB19 | IncFIB(K) IncFII(K) |
| 13 |  | aph(3')-VIb aph(3'')-Ib aph(6)-Id aac(6')-Ib | gyrA_S83I parC_S80I qnrS | emrD oqxA oqxB | ColKP3 IncA/C2 IncFIB(K) IncFII(K) IncX3 |
| 14 | msr(E) mph(E) | armA | gyrA_S83I parC_S80I | emrD oqxA oqxB20 | Col156 Col(MG828) ColRNAI IncFIA(HI1) IncFIB(K) IncFII(K) IncR |
| 15 | msr(E) mph(E) | armA | gyrA_S83I parC_S80I | emrD oqxA oqxB20 | ColRNAI IncFIA(HI1) IncR |
| 16 | mph(A) | aadA2 | gyrA_S83I parC_S80I | emrD oqxB | IncFIB(K) IncFIB(pQil) IncFII(K) IncX3 |
| 17 | msr(E) mph(E) | armA aph(3'')-Ib aph(6)-Id aadA1 | gyrA_S83I parC_S80I | emrD oqxA oqxB20 | Col156 Col440II ColRNAI IncFIA(HI1) IncFIB(AP001918) IncFII IncFII(K) IncR IncQ1 |
| 18 | msr(E) mph(E) | armA | gyrA_D87N,gyrA_S83Y parC_S80I | emrD oqxA oqxB20 | ColRNAI IncFIA(HI1) IncR |
| 19 | msr(E) mph(E) | armA | gyrA_D87N,gyrA_S83Y parC_S80I | emrD oqxA oqxB20 | Col156 Col(MG828) ColRNAI IncFIA(HI1) IncFII(K) IncR |
| 20 | msr(E) mph(E) | armA aac(3)-IIe aac(6')-Ib-cr5 | gyrA_D87N,gyrA_S83Y parC_S80I | emrD oqxA oqxB20 | ColRNAI IncFIA(HI1) IncFIB(K) IncFII(K) IncR |
| 21 | mph(A) | aph(3')-Ia aadA2 aac(6')-Ib | gyrA_S83I parC_S80I | emrD oqxA oqxB | ColRNAI IncFIB(K) IncFIB(pQil) IncFII(K) |

**Figure S1.** *In vitro* acitvity of cefepime (FEP), cefepime/enmetazobactam (FPE) and cefepime/zidebactam (FPZ) against all KPC-producing Klebsiella pneumoniae included in this study





**Table S2.** Sequence insertions/deletions and substitutions of different KPC variants included in this study compared to KPC-2

| **KPC** | **Mutations compared to KPC-2** |
| --- | --- |
| KPC-3 | H237Y |
| KPC-14 | G241_DGT_A244 |
| KPC-31 | D179Y, H273Y |
| KPC-33 | D179Y |
| KPC-49 | R164S, H273Y |
| KPC-93 | N268_ins(NRAPN)_K269 |
| KPC-167 | D179Y, K269_ins(DDKYSE)_D170 |
| KPC-203 | E166_DLE_L169, A261_ins(VYTRAPMLA)_V262 |
| KPC-205 | N268_ins(NRAPN)_K269, H273Y |

**Table S3**. Antimicrobial susceptibility profiles of the KPC-producing *Klebsiella pneumoniae* clinical strains included in this study

| **Strain** | **KPC Variant** | **MIC**  **(mg/L)** | | | | |
| --- | --- | --- | --- | --- | --- | --- |
|  |  | **Cefepime** | **Cefepime/Enmetazobactam** | **Cefepime/Zidebactam** | **Avibactam/Aztreonam** | **Ceftazidime/Avibactam** |
| 1 | KPC-31 | 12 | 0.38 | 0,125 | 0.38 | 48 |
| 2 | KPC-31 | 12 | 0.38 | 0,047 | 0.5 | 48 |
| 3 | KPC-31 | 12 | 0.5 | 0,064 | 0,25 | 32 |
| 4 | KPC-3 | >=64 | >=64 | 0,5 | 1 | 8 |
| 5 | KPC-3 | 12 | 0,047 | 0,38 | 0.47 | 64 |
| 6 | KPC-3 | >=64 | >=64 | 0,064 | 0.19 | 3 |
| 7 | KPC-205 | 48 | 1.5 | 0,094 | 0.75 | 64 |
| 8 | KPC-203 | 4 | 0.75 | 0,094 | 0,064 | 16 |
| 9 | KPC-3 | >=64 | >=64 | 0,38 | 0,5 | 3 |
| 10 | KPC-3 | >=64 | >=64 | 0.38 | 0.19 | 1.5 |
| 11 | KPC-2 | 16 | 125 | 0.5 | 125 | 1 |
| 12 | KPC-3 | >=64 | >=64 | 1 | 0,75 | 3 |
| 13 | KPC-93 | >=64 | >=64 | 0,064 | 2 | >256 |
| 14 | KPC-2 | >=64 | >=64 | 0,5 | 0.5 | 1.5 |
| 15 | KPC-49 | 24 | 1 | 0,38 | 1 | 16 |
| 16 | KPC-2 | >=64 | 24 | 0,125 | 0,125 | 0,5 |
| 17 | KPC-33 | 12 | 0,75 | 0,125 | 0,125 | 8-16 |
| 18 | KPC-14 | 32 | 0,75 | 0,016 | 0,75 | >=64 |
| 19 | KPC-14 | 64 | 0.75 | 0,094 | 3 | >256 |
| 20 | KPC-3 | >=64 | >=64 | 0.125 | 0,5 | 4 |
| 21 | KPC-167 | >=64 | 0.75 | 0.19 | 0.38 | >256 |
